# Maternal Wnt/β-Catenin Signaling Coactivates Transcription through NF-κB Binding Sites during Xenopus Axis Formation

**DOI:** 10.1371/journal.pone.0036136

**Published:** 2012-05-09

**Authors:** Neil J. Armstrong, François Fagotto, Christian Prothmann, Ralph A. W. Rupp

**Affiliations:** 1 Department of Molecular Biology, Adolf-Butenandt-Institute, Ludwig-Maximilians-University, Munich, Germany; 2 Department of Biology, McGill University, Montreal, Quebec, Canada; University of Massachusetts Medical School, United States of America

## Abstract

Maternal Wnt/β-Catenin signaling establishes a program of dorsal-specific gene expression required for axial patterning in Xenopus. We previously reported that a subset of dorsally expressed genes depends not only on Wnt/β-Catenin stimulation, but also on a MyD88-dependent Toll-like receptor/IL1-receptor (TLR/IL1-R) signaling pathway. Here we show that these two signal transduction cascades converge in the nucleus to coactivate gene transcription in blastulae through a direct interaction between β-Catenin and NF-κB proteins. A transdominant inhibitor of NF-κB, ΔNIκBα, phenocopies loss of MyD88 protein function, implicating Rel/NF-κB proteins as selective activators of dorsal-specific gene expression. Sensitive axis formation assays in the embryo demonstrate that dorsalization by Wnt/β-Catenin requires NF-κB protein activity, and vice versa. Xenopus nodal-related 3 (Xnr3) is one of the genes with dual β-Catenin/NF-κB input, and a proximal NF-κB consensus site contributes to the regional activity of its promoter. We demonstrate in vitro binding of Xenopus β-Catenin to several XRel proteins. This interaction is observed in vivo upon Wnt-stimulation. Finally, we show that a synthetic luciferase reporter gene responds to both endogenous and exogenous β-Catenin levels in an NF-κB motif dependent manner. These results suggest that β-Catenin acts as a transcriptional co-activator of NF-κB-dependent transcription in frog primary embryonic cells.

## Introduction

In vertebrates, canonical Wnt/β-Catenin signaling plays a key role in early embryonic patterning. This role is probably best understood in the frog Xenopus laevis, where fertilization triggers a rotation of the egg cortex leading to subsequent accumulation of nuclear β-Catenin over a broad territory on the prospective dorsal side of the embryo [1]. Loss of function experiments have indicated that signaling through maternal Wnt11 stabilizes β-Catenin, which converts repression of specific target genes through Tcf transcription factors into transcriptional activation [2], [3], [4]. While the molecular details of this switch are not fully understood, β-Catenin antagonizes binding of groucho/CtBP co-repressor complexes and promotes gene activation through recruitment of chromatin remodeling factors and other transcriptional regulators such as VegT and FoxH1 [4], [5]. Nuclear β-Catenin activity is required for gene activation in several dorsally located signaling territories, including at blastula the dorsovegetal Nieuwkoop Center and the dorsoanimal chordin noggin expressing center (BCNE), followed at gastrula by Spemann's Organizer in the marginal zone [6]. Their distinct temporal and biochemical activities orchestrate embryonic patterning in the forming germ layers and body axes.

Maternal Wnt/β-Catenin activity is essential for axial patterning in vertebrates. However, it is not sufficient and requires interaction with other pathways, e.g. with VegT and Nodal/TGF-β signals to initiate mesendoderm formation [5], [7]. We have previously shown that interference with maternal XMyD88 protein function soon after fertilization impairs axis formation and selectively reduces expression of pivotal regulatory genes [8]. MyD88 is a conserved adaptor protein, which is devoted to a specific branch of NF-κB activating pathways, which are triggered through TLR/IL-1R signaling [9]. This pathway is well known for its role in the innate immune response, both in vertebrates and insects [10]. Initial studies in Drosophila identified the prototypic Toll receptor as part of a maternal signaling cascade, which establishes dorsoventral patterning of the embryo through signal dependent degradation of Cactus, a member of the IκB protein family, thus facilitating nuclear uptake of the Dorsal transcription factor, a member of the Rel/NF-κB protein family [11]. In early development, the ligand of the fly Toll receptor is locally produced by proteolytic processing of the Spätzle precursor protein through the protease Easter [12], [13]. This pathway has been conserved to some extent in vertebrates, since misexpression of Drosophila Easter and Spätzle can partially rescue dorsal axial structures in UV-ventralized Xenopus embryos in a XMyD88 dependent manner [8], [14]. These results imply NF-κB proteins as important regulators of early gene expression programs in the frog. Due to its selective effects on dorsally expressed genes, this pathway is likely to cooperate in some way with maternal Wnt/β-Catenin signaling.

Several members of the Rel protein family are expressed broadly both as maternal and zygotic transcripts in Xenopus embryos, including Xrel1/XrelA/p65 [15], [16], XrelB [17], Xrel2 [18], Xrel3 [19], and p100/p52 [20]. Furthermore, Xenopus oocytes contain a deoxycholate-inducible NF-κB-like activity, containing a subunit which crossreacts with p105/p50-specific antibodies [21]. In situ, cytoplasmic Xrel1/XrelA/p65 protein is detectable in an animal to vegetal gradient in cleavage embryos, and translocates to the nucleus around the midblastula [22]. Thus, Rel proteins are present in the frog from fertilization onwards and appear to become activated concomitant with the onset of zygotic transcription.

Antisense morpholino oligonucleotides offer a convenient method for loss-of-function studies in Xenopus. However, since morpholinos inhibit the translation of mRNAs but have no effect on preexisting protein, we decided to examine the developmental functions of Xenopus Rel proteins via a different approach. We employ a transdominant NF-κB inhibitor, i.e. ΔNIκBα, which lacks the first 40 amino acids containing the critical serine residues involved in its signal-dependent degradation. Forced expression of ΔNIκBα has been shown to antagonize nuclear translocation of NF-κB complexes [23] and to cause loss of NF-κB function phenotypes in chick and zebrafish [24], [25].

We report here that in Xenopus, ΔNIκBα causes virtually the same phenotype as inhibition of MyD88 protein function, i.e. a preferential loss of anterior head structures due to reduced expression of a specific subset of dorsally expressed genes. The dnMyD88/ΔNIκBα-sensitive genes are known to depend on maternal Wnt/β-Catenin signaling, suggesting a close interaction between the two pathways. This assumption is verified by three independent experimental settings, which demonstrate that dorsal axis formation by Wnt/β-Catenin requires NF-κB activity, and vice versa. On the mechanistic level, we show that NF-κB consensus DNA binding sites in the context of a synthetic reporter construct sense and respond positively to enhanced nuclear β-Catenin protein levels. Furthermore, co-immunoprecipitation experiments indicate a direct interaction between β-Catenin and XrelA, which is observed in Wnt-stimulated cells. Together, these results suggest for the first time, a direct coactivation of NF-κB-dependent transcription through canonical Wnt/β-Catenin signaling.

## Results

### NF-κB activity is required for axis formation

Our previous analysis implicated TLR/IL-1 receptor signaling in expression of dorsal-specific genes and in head formation. This insight was derived from interference analysis, in which overexpression of the Toll/IL-1 homology (TIR) domain of MyD88 was used to specifically uncouple TLR/IL-1 receptor signaling from NF-κB activation near the cell membrane (see [8] and references therein). Here, we extend this analysis by using a transdominant IκBα variant to investigate the consequences of blocking the same pathway at the level of the transcription factor NF-κB. ΔNIκBα, in which the N-terminal sequences responsible for signal-dependent degradation are missing, is a potent inhibitor of Rel/NF-κB function in vivo [24], [26]. We were interested to see what effect this transdominant inhibitor molecule would have if used to block NF-κB activity during early Xenopus embryogenesis. To do so we injected RNA encoding ΔNIκBα into targeted regions of the embryo over a range of time points and doses.

**Figure 1:**
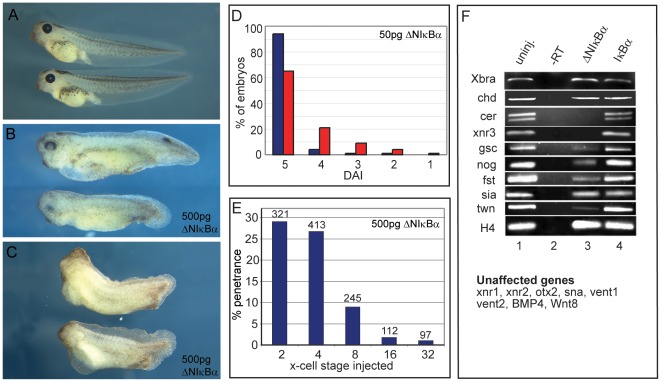
NF-κB activity is required for head formation. **A**) Wild type embryos at stage 40/41. **B, C**) Sibling embryos injected sub-equatorially on the dorsal side with 500 pg of ΔNIκBα RNA at the 4-cell stage show pleiomorphic phenotypes: B) Mild phenotypes (DAI 4) are characterized by reduction in forebrain and eyes. C) Stronger phenotypes (DAI 3) show reduced head and dorsal structures. (**D**) Graphical representation of dorsoanterior index (DAI) of control embryos (blue columns) versus embryos injected dorsally with 50 pg of ΔNIκBα (red columns). (**E**) Time course analysis of embryos injected dorsally with a total of 500 pg ΔNIκBα RNA at the two, four, eight, 16- and 32-cell stage; the accompanying decrease in cell size was compensated by injecting multiple dorsal cells from the 8-cell stage on (see materials and methods). Y-axis represents the percentage of embryos with anterior truncations. (**F**) Semi-quantitative RT-PCR analysis for marker genes in stage 10 marginal zone explants. Lane 1: uninjected embryos; lane 2: minus reverse transcriptase control; lane 3: 500 pg ΔNIκBα RNA injected dorsally; lane 4: 500 pg wildtype IκBα RNA injected dorsally.

We first tested whether ΔNIκBα would affect formation of normal dorsal-anterior structures, a sensitive assay to detect interference with dorsalizing signals. The strength of these signals is reflected phenotypically after hatching by a series of characteristic anatomical defects, which are scored on a scale called the dorsoanterior index (DAI, [27]). When the signal is increased, “hyperdorsalized” embryos are formed (DAI>5), which display exaggerated head structures paralleled by disappearance of posterior structures. When the signal is decreased, “ventralized” embryos are obtained (DAI<5), which lack dorsal and anterior structures. 50 pg (n = 415) or 500 pg (n = 233) of ΔNIκBα RNA were injected into the dorsal blastomeres of the 4-cell stage embryo. Approximately one third of the embryos injected with the low dose showed dorso-anterior reduction in head structures (Figure 1D). The majority of these correspond to a DAI score between 3 and 4 (i.e. reduced eyes/forehead or cyclopia). About a quarter of these embryos were more severely affected, being scored as 1 and 2 (micro- and acephalic). No significant difference was observed between the low and high dose of ΔNIκBα RNA, and representative examples of the described weak and strong phenotypes from embryos injected with the high dose of ΔNIκBα are shown in Figure 1B and C. This suggests that maximal inhibition of NF-κB activity by this method has been achieved. To control that the injection itself was not responsible for the effects, sibling embryos were injected with distilled water alone, 250 pg lacZ RNA or 500 pg wildtype IκBα. In all cases, no significant differences were seen compared to non-injected embryos (data not shown).

To test whether timing of the injection played a role, 500 pg ΔNIκBα RNA was injected sub-equatorially into the dorsal side of embryos from the 2-cell to the 32-cell stage. To ensure that the same region was covered by mRNA under all conditions, multiple cells were injected for the 16- and 32-cell stage (see materials and methods). As shown in Figure 1E, maximal penetrance for the dorso-anterior reduction phenotype was observed for injections at the two and four-cell stages (average: 27%, n = 734). Notably, the penetrance fell three-fold at 8-cell stage injections (9%, n = 245) and a further three-fold at the 16-cell stage (3%, n = 112), only slightly higher than non-injected sibling embryos. Injections at the 32-cell stage had no significant effect. Even if one considers that it takes time to build up protein levels from RNA injection, ΔNIκBα has to interfere very early with development to achieve this effect. For this reason, injections were performed at the 2–4-cell stage in subsequent experiments.

We next analyzed the expression of potential gene targets in marginal zone explants of dorsally injected versus non-injected embryos (Figure 1F). Of the 18 genes tested, six genes were downregulated by the injection of ΔNIκBα: Cerberus (cer) and nodal-related 3 (Xnr3) were ablated; goosecoid (gsc), twin (twn), follistatin (fst) and noggin (nog) were reduced. Two other dorsal markers, siamois (sia) and chordin (chd) were unaffected, and also panmesodermal (xbra) or ventral (vent-1, vent2, bmp4, wnt8) marker genes were neither up nor down regulated. We conclude from this that NF-κB is required for activation of a specific subset of dorsally expressed genes.

Notably, the results with ΔNIκBα were virtually identical to those produced by dominant negative XMyD88 [8] with regard to the morphological phenotype, the temporal requirements for interference, and the specific subgroup of target genes. Together, these studies strongly imply a requirement for TLR/IL1-R/MyD88/IκBα-mediated activation of maternal NF-κB protein very early in development. In addition, they suggest a close link between WNT and TLR/IL1-R signaling pathways, which we have investigated further.

### β-Catenin and NF-κB cooperate in secondary axis formation

**Figure 2:**
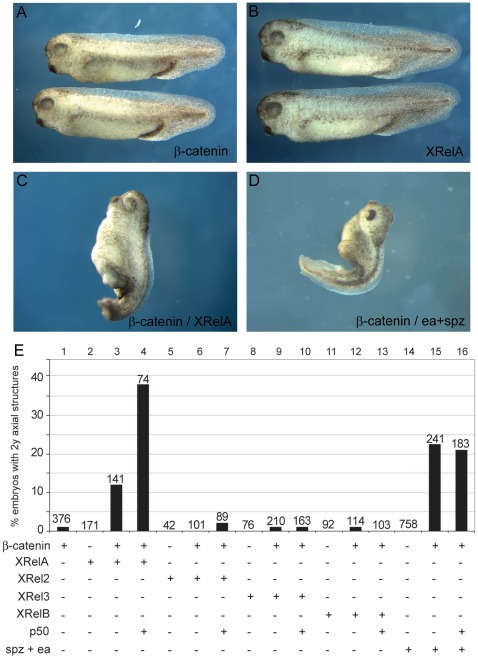
Synergism of XRel and β-Catenin proteins in ectopic axis formation. Suboptimal levels of β-Catenin synergize with XRel proteins or Drosophila Easter/Spätzle to induce axial structures on the ventral side. Embryos injected ventrally with low levels (4 pg) of β-Catenin (**A**) or 500 pg of XRelA (**B**), XRel2, XRel3, XrelB RNAs were indistinguishable from uninjected sibling embryos. Co-injection of 500 pg XRelA with 3 pg of β-Catenin could induce rudimentary axial structures (**C**), as could co-injection of 100 pg each of easter and spätzle with 3 pg of β-Catenin (**D**). Graphical representation (**E**) of all injected combinations, indicating a selective synergism between ectopic β-Catenin and exogenous XrelA/p50 heterodimers, as well as with endogenous NF-κB proteins released by ectopic Ea/Spz.

Injection of β-Catenin RNA into the ventral side of early Xenopus embryos can be sufficient to induce a complete duplication of dorsal and anterior structures. We performed dose titration experiments to determine the threshold concentration above which β-Catenin RNA starts to induce secondary axes. Under our experimental conditions, a dose of 4 pg of β-Catenin RNA still produced embryos (Figure 2A) that were indistinguishable from uninjected sibling embryos. Similarly, ventral injections of 500 pg of XrelA alone (Figure 2B) did not induce axial structures, the same being true for 500 pg of XRel2, XRel3 and XrelB RNAs (Figure 2E). However, 500 pg of XRelA co-injected with 3 pg of β-Catenin resulted in the creation of short axial protrusions in 12% of the injected embryos (Figures 2C and E). Cross-sections of these truncated axes revealed the presence of neural tissue and muscle, but no notochord (data not shown). Significantly, co-injection of p50 together with this combination was able to increase the penetrance, although not the quality, of the phenotype 3-fold (39%, n = 74). The most frequently found activated form of NF-κB in TLR/IL-1 signaling is a RelA/p50 heterodimer [28], and this effect may indicate a similar preference for Xenopus embryos. In contrast, co-injections of β-Catenin with XRel2, XRel3 or XRelB did not produce such phenotypes (Figure 2E), implying a selective synergism between XrelA (and p50) with Wnt-stimulated β-Catenin.

We next wanted to know whether this synergism was also apparent under more physiological stimulation conditions, involving signaling through TLR receptors rather than overexpresssion of Rel proteins. The ligand which is responsible for the MyD88/IκBα-sensitive NF-κB activation in the frog embryo is unknown. Therefore, we injected RNA for Drosophila easter and spätzle proteins, which we have previously shown to elicit a dorsalizing activity in UV-ventralized Xenopus embryos, which is blocked by a transdominant variant of Cactus, the fly IκBα homolog [14]. The combined activities of Ea and Spz alone were insufficient, but in combination with a sub-threshold β-Catenin dose, this combination produced small secondary axes (Figure 2D and E). The frequency was actually higher than for the combination of RelA and β-Catenin but the coinjection of p50 had no additional effect (Figure 2E, compare lanes 3 and 15–16). Together, we conclude from these experiments that the axis-inducing activity of β-Catenin is enhanced through a selective synergism with either exogenous RelA, exogenous RelA/p50 heterodimers, or Ea/Spz-induced endogenous NF-κB complexes.

### Axis duplication by ectopic β-Catenin depends on endogenous NF-κB activity

**Figure 3:**
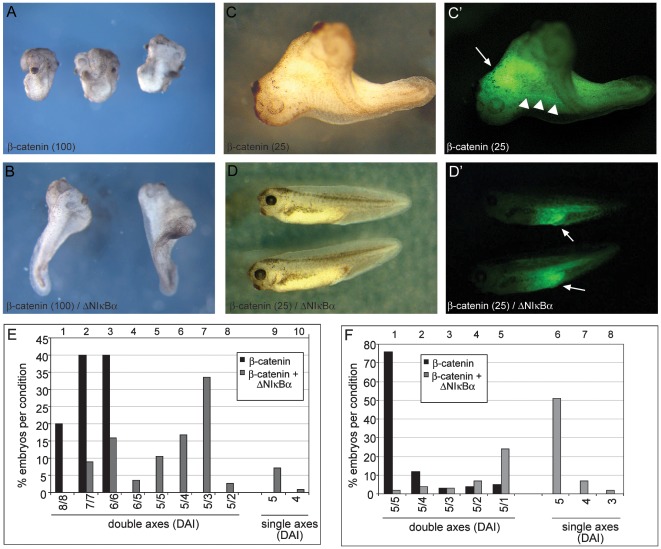
Dorsalisation by ectopic β-Catenin depends on endogenous NF-κB activity. Embryos were injected ventrally with high doses (100 pg, panels **A,B**) or low doses (25 pg **C,D**) of β-Catenin RNA, alone (**A,C**), or with 500 pg ΔNIκBα RNA (**B,D**). All injections included GFP RNA for lineage tracing (C′,D′). **A**) Embryos injected with high β-Catenin RNA were highly dorsalized (average DAI 6.8 for each axis), almost always lacking posterior structures (n = 160). **B**) Co-injection of 500 pg of ΔNIκBα reduced the secondary axis to an average DAI = 3.98, with a small number (8%) being single axis embryos (n = 210). **C**) The majority of embryos injected with low β-Catenin RNA produced pairs of complete axes, both of which scored as “5” on the DAI scale (n = 155). **D**) Coinjecting 500 pg of ΔNIκBα produced mostly single axis embryos (n = 162). (**C′,D′**) Lineage tracing showed that the β-Catenin RNA-injected cell progeny accumulated in the anterior region of one of the two axis (white arrow) with some posterior trailing in axial tissue (white arrowheads) (**C′**), while it was localized in posterior ventral tissues when ΔNIκBα was co-injected (**D′**). Panels **E** and **F** summarize the phenotypic penetrance for respectively high (100 pg) and low (25 pg) β-Catenin RNA (3 independent experiments in each case). Numbers on the X-axis indicate DAI-values of each embryonic Anlage (e.g. 5/5 defining a twinned embryo with two complete heads).

Next we examined whether the presence of endogenous, nuclear NF-κB was required for the ability of β-Catenin to induce secondary axes. For this purpose, we provided first saturating amounts of ectopic β-Catenin activity. Ventral injection of 100 pg β-Catenin RNA created hyperdorsalized embryos, consisting of multiple heads and often lacking tail structures (Figure 3A). When this high dose of β-Catenin was co-injected with ΔNIκBα, the quality of the secondary axis formation was severely reduced (Figure 3B). Indeed, the average DAI score of the double axes was reduced from 6.8 to 3.98 (Figure 3E, compare black with grey columns). Furthermore, in 8% of the cases, no secondary axes were formed at all (ibid., lanes 9 and 10).

A lower dose of β-Catenin RNA (25 pg) injected ventrally was enough to induce a secondary axis with a complete head, but without affecting posterior tissues (see Figure 3C). Such secondary axes were scored as 5 (“normal” axis) on the DAI scale and made up the majority of embryos injected with this lower dose of β-Catenin (Figure 3F). Co-injection of ΔNIκBα RNA led to a significant quantitative and qualitative reduction of secondary axes formed. In fact, the majority of embryos returned to a single axis state under these conditions (Figure 3D and F, compare black versus grey columns). GFP lineage tracing shows that the progeny of the injected blastomeres acquire specific positions within the embryo dependent on the experimental condition. In cases of complete secondary axes, green cells localize to the anterior endomesoderm with some posterior trailing along midline tissues (Figure 3C′), consistent with their “Organizer” activity. In cases where second axis formation by β-Catenin was inhibited through ΔNIκBα, green cells are found at a ventroposterior position around the proctodeum (Figure 3D′), corresponding to the normal fate of cells derived from the ventral site of injection. The cells' position, therefore, is correlated to their amount of dorsalizing activity, indicating that they are viable and capable of populating different embryonic regions. Based on the observed antagonism, we conclude that ΔNIκBα is capable of blocking β-Catenin function during secondary axis formation, most likely through the observed interference with the expression of co-regulated target genes (see Figure 1). This effect is remarkably strong, since significant inhibition is observed even in the presence of very large amounts of β-Catenin mRNA. In contrast, formation of the primary body axis seems to be less sensitive to dorsal injection of ΔNIκBα mRNA (see Figure 1D), perhaps because translation of the inhibitory protein comes too late to interfere as strongly with the endogenous dorsalizing signal.

### Dorsalizing activity of Easter/Spätzle in Xenopus depends on β-Catenin

As shown above (Figure 2E, lane 14), injection of Ea and spz RNAs is insufficient to induce dorsalization of the ventral side in normal embryos. UV-ventralized embryos, in which cortex rotation has been blocked and, thus, nuclear β-Catenin is observed in the vegetal pole area rather than on the prospective dorsal side of the embryo [1], represent a sensitized system for axis induction. Indeed, we have shown that Ea/spz can rescue dorsal structures such as a segmented musculature and neural tissue in fully ventralized UV-treated embryos, although never producing a full axis [14]. These findings prompted us to investigate whether the NF-κB activity released through Ea/spz required the presence of nuclear β-Catenin. To address this question, we make use of an EP-cadherin variant, from which the extracellular domain had been deleted (EP-cadΔE). This variant efficiently sequesters soluble β-Catenin at the cell membrane and, thus, blocks canonical Wnt signaling to the nucleus [29], [30].

**Figure 4:**
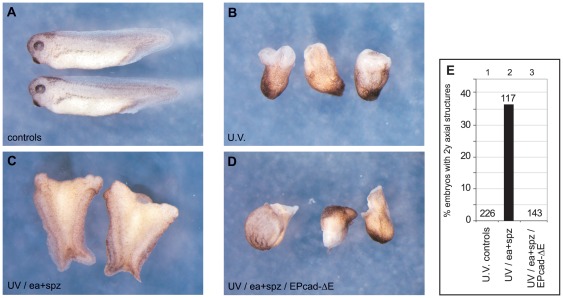
ea/spz-dependent dorsalization requires β-Catenin activity. (**A**) Untreated controls at tadpole stage (NF 35/36). (**B**) UV-ventralized siblings (average DAI = 0.30). (**C**) UV-ventralized embryos injected sub-equatorially with 100 pg each of Drosophila easter and spätzle RNA produced embryos with 2 rudimentary trunk axes (37%). (**D**) Embryos injected with 50 pg EPcadΔE, in addition to easter and spätzle, did not show any axial rescue (0%). (**E**) Graphical representation of axial rescue, giving total numbers for each experimental cohort.

Xenopus embryos were ventralized by UV-irradiation, producing embryos with an average DAI of 0.21 (“Bauchstück”, i.e. maximally ventralized; n = 226) (Figure 4B). Control, sibling embryos had a DAI of 4.9 (Figure 4A). Injection of a mixture of activated easter (EaΔN) and spätzle RNAs sub-equatorially into opposing blastomeres of the ventralized embryos produced double axes in 37% of cases (n = 117, Figure 4C) – the double axes being proof that the two opposing injection sites function as dorsalizing centers - and raised the average DAI to 2.02 (microcephalic). Co-injection of EP-cadΔE RNA prevented this rescue of dorsal structures (Figure 4D, n = 143) producing embryos with an average DAI of 0.30 – comparable to that of the UV-ventralized controls. These results provide a third experimental line of evidence that maternal Wnt signaling and a TLR/IL1-R/NF-κB pathway functionally interact during axis formation.

### The Xnr3 promoter receives input from MyD88/NF-κB pathway through a single consensus κB-site

One of the genes responding strongly to inhibition of NF-κB function through interference with MyD88 and IκBα is *nodal related 3* (Xnr3; Figure 1 and [8]). It encodes a TGFβ-related family member, expressed in the epithelial cell layer of the organizer and displaying complex functions, including sequestration of BMP ligands in the extracellular space [31], [32], [33]. The Xnr3 promoter contains a well-characterized Wnt-response element, called WRE2, with a consensus Tcf/Lef binding site [34], thought to be bound by Tcf/Lef/β-Catenin complexes during Wnt-induced transcription [35].

**Figure 5:**
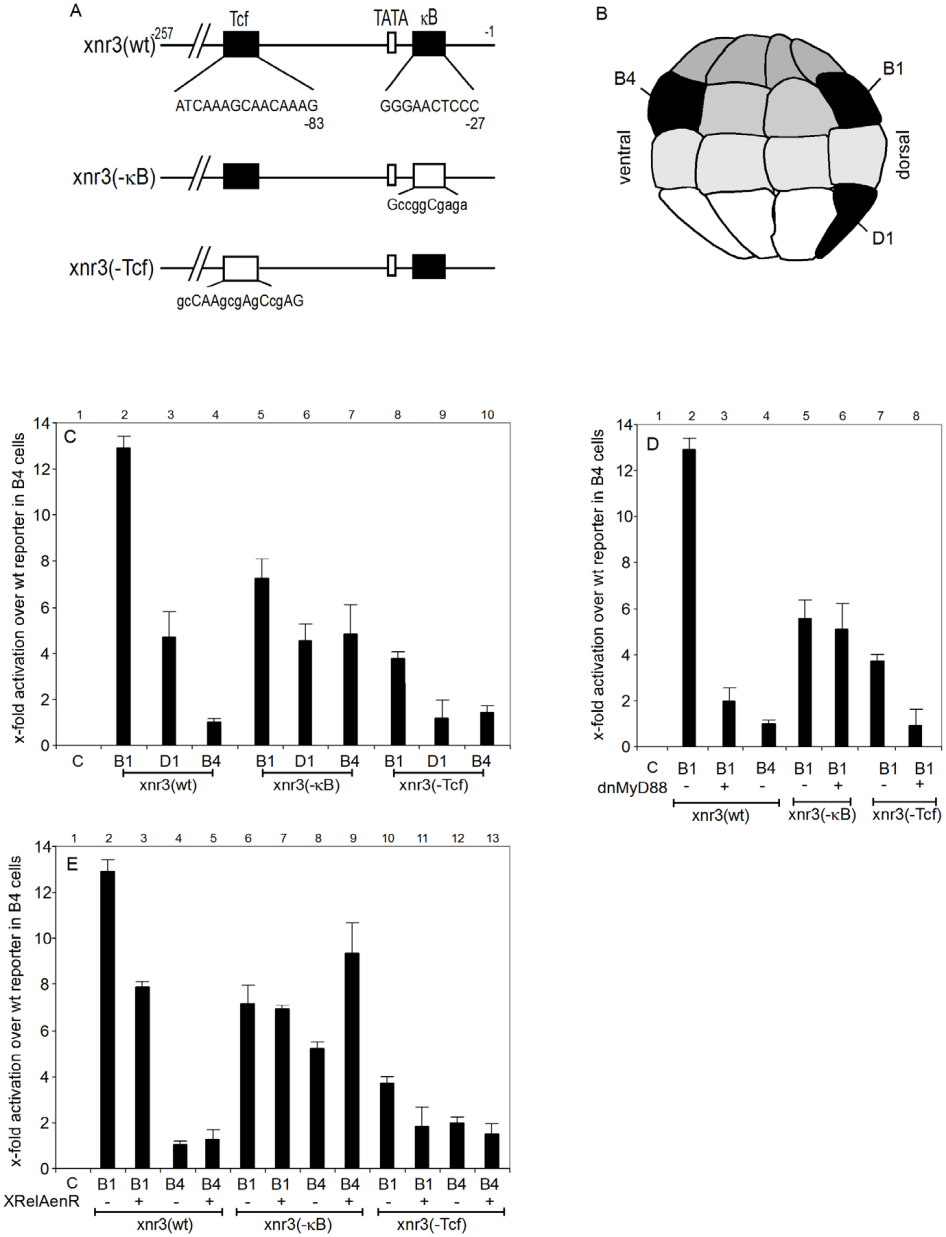
The Xnr3 promoter is regulated through a consensus NF-κB DNA binding site. Panel (**A**) - Pictorial representation of the Xnr3 promoter and its κB and Tcf binding sites, as well as representations of mutated promoters, which were used in the luciferase reporter gene assays. Bases at mutated nucleotide positions are shown in lower-case letters. (**B**) Lateral schematic view of a 32-cell stage embryo locating the B1, B4 and D1 blastomeres in black, whose progeny will later contribute to the BCNE, the ventral gastrula center (VGC) and the Nieuwkoop center (NC), respectively. (**C**) Results of injection of a group of wildtype and mutant Xnr3 promoter driven luciferase constructs, each injected into the B1, B4 and D1 blastomeres of 32-cell stage embryos; y-axis describes x-fold production of luciferase, normalized to an internal renilla control, relative to the luciferase activity of uninjected embryos (lane 1). (**D**) and (**E**) Luciferase activities of wildtype and mutant Xnr3 reporter constructs, injected into B1 or B4 blastomeres as indicated, under conditions, in which endogenous NF-κB activity is either antagonized through coninjected XrelAenR (**D**) or reduced through dominant-negative XMyD88 (**E**). Luciferase activity was measured at midgastrula (NF11).

We now re-examined the Xnr3 promoter sequence and discovered an NF-κB consensus site [36] upstream of position −27, in between the TATA box and the transcriptional start site (Figure 5A). We generated three luciferase reporter constructs, which contained DNA sequences up to −257, i.e. the same region used before to characterize the Wnt-responsiveness of this promoter [34]. These included a wildtype promoter [Xnr3(wt)], and two mutant versions, Xnr3(−κB) and xnr(−Tcf), in which the κB and Tcf/Lef sites have been destroyed by mutating critical nucleotide positions (Figure 5A). The activities of these plasmids in Xenopus embryos were then tested by targeted injection into either dorsal vegetal (D1), dorsal animal (B1) or ventral animal (B4) blastomeres of the 32-cell stage embryo (Figure 5B). These injection sites correspond to important territories - the Nieuwkoop center, the BCNE and the ventral gastrula center, respectively - which control body axis formation in Xenopus [6].

As expected from the expression profile of Xnr3, luciferase activity was strongest in B1-injected embryos, reduced by ∼2.5 fold in D1 injections, and it was minimal in ventral B4 cells (Fig. 5C, lanes 2–4). When the κB site was mutated, activity in B1 was reduced, indicating that this site stimulates Xnr3 transcription in the BCNE. Luciferase activity in D1 was unaffected, while in B4 it was up-regulated (ibid, lanes 5–7). The results for this mutant on the dorsal side are consistent with the reported animal to vegetal gradient of XRelA protein at blastula [22], while its increased activity in B4 suggests that this site exerts a repressive influence on Xnr3 promoter in the territory of the ventral gastrula center.

Luciferase activity of the Xnr3(−Tcf) construct was reduced in the Nieuwkoop Center (D1) down to the basal levels found in the ventral gastrula center (B4; panel C, compare lanes 9 and 10), confirming previous results on the importance of the WRE2 element [34]. However, this mutation had a much weaker effect in the BCNE (B1, lane 8), where despite an approximately three-fold reduction compared to the wildtype construct, it still showed a significant residual activity (compare lanes 2 with 8–10). This residual activity could either be provided through the NF-κB site or other promoter elements. To distinguish between these possibilities, we tested the reporter constructs under different NF-κB loss-of-function conditions.

In a first experimental series, we co-injected RNA encoding a dominant-negative fusion protein, consisting of the engrailed transcriptional repressor domain and the rel homology domain (RHD) of the XRelA protein. RHDs mediate dimerization, nuclear localization and DNA-binding [37]. This fusion protein is expected to compete with endogenous NF-κB complexes and repress their target genes. As shown in Figure 5D, XrelAenR reduced luciferase expression from both wildtype and WRE2-mutated reporter genes in the BCNE (B1; compare lanes 2–3, and 10–11), but had no effect on the Xnr3(−κB) reporter construct (compare lanes 6–7). This indicates that repression of luciferase activity by XrelAenR requires the presence of an NF-κB binding site on the reporter gene. Interestingly, in the ventral gastrula center (B4), where the deletion of the NF-κB motif derepressed the wildtype reporter construct (panel C, lanes 4 and 7), XrelAenR increased the activity of the Xnr3(−κB) reporter (Panel D, lanes 8 and 9). The activity of the Xnr3(−κB) reporter in B4 progeny could involve nuclear β-Catenin, which appears on the ventral side of the embryo at the blastula/gastrula transition [38]. Its hyperactivation, however, must be an indirect effect, possibly involving repression of endogenous bmp transcription by XrelAenR (Armstrong and Rupp, unpublished result).

In a second experimental series, we analyzed the consequences of blocking NF-κB activation through dominant-negative MyD88, which uncouples TLR/IL1-receptor signaling from IKK-dependent IκB degradation [8], [9]. The results from this series (see panel E) were very similar to the effects obtained with the dominant-negative XrelAenR protein. This included a strong reduction of luciferase expression in the BCNE for both wildtype and xnr3(−Tcf) reporter genes (compare lanes 2 and 3, and lanes 7 and 8), apparent only in the presence of the NF-κB binding site (lanes 5 and 6). These results imply that both wt and −Tcf reporter genes, but not the −κB construct, are activated through endogenous NF-κB protein complexes.

Taken together, this reporter gene analysis suggests a dual function for NF-κB protein complexes in transcriptional regulation of the Xnr3 promoter. Maximal Xnr3 expression in the BCNE requires activation through Rel/NF-κB protein complexes binding to a consensus NF-κB DNA binding site near the TATA box, in addition to stimulation through the upstream WRE2 element. The lower Xnr3 promoter activity on the ventral side of the embryo results in part from the absence of maternal Wnt/β-Catenin activity and possibly from repression through the NF-κB motif. Although XrelA/p65-containing NF-κB complexes can actively repress genes [39], an involvement of non-Rel proteins targeting the vicinity of the NF-κB binding site in the ventral gastrula center cannot be ruled out. Most importantly, our results provide compelling evidence for a convergence of maternal WNT/β-Catenin and MyD88-sensitive TLR/IL1-receptor signaling pathways on the promoter of the Xnr3 target gene.

### Rel proteins form complexes with β-Catenin

In principal, Wnt and TLR/IL1-R pathways may cooperate in Xnr3 transcription through the neighboring Tcf and κB sites or converge on the κB motif through an interaction between DNA-bound NF-κB protein complexes and nuclear β-Catenin. Evidence for an indirect interaction of β-Catenin with NF-κB, apparently requiring an unknown cellular factor comes from cancer cell lines; unlike in our case, however, interaction with β-Catenin inhibits NF-κB mediated gene transcription in this context [40], [41].

**Figure 6:**
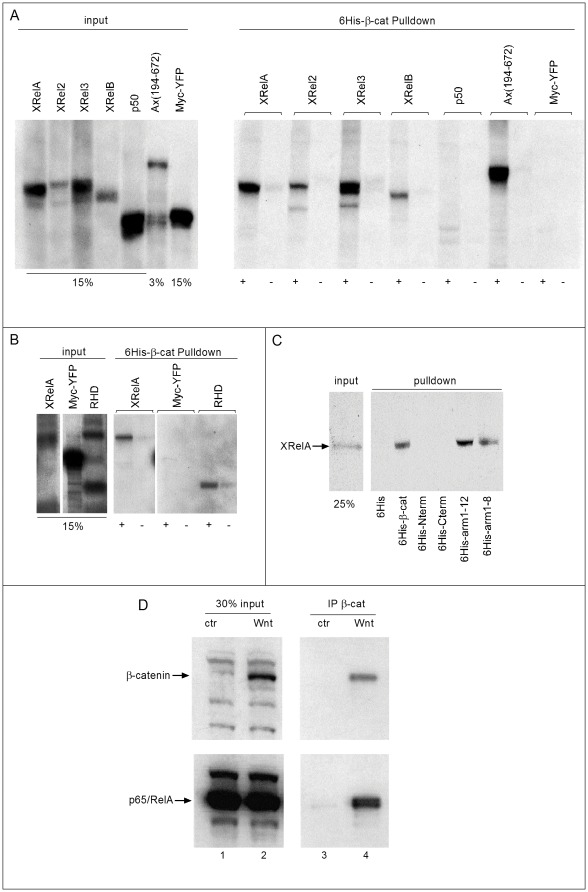
Interaction of β-Catenin and Rel/NF-κB proteins. **A–C**: in vitro pull down using his-tagged bacterial recombinant β-Catenin and ^35^S-labelled in vitro translated Rel proteins. (**A**) XrelA, 2, 3 and B, but not p50, co-precipitated with full length β-Catenin fused to 6His-Trx-S-tag, but not with a control 6His-Trx-S-tag fragment (Tag abbreviated as “6His” in panels). Ax(194–762), an Axin fragment containing the β-Catenin binding domain and Myc-YFP were used as positive and negative controls for the in vitro translated proteins, respectively (n≥3 independent experiments). (**B**) XRelA interacts with β-Catenin via its RHD domain. (**C**) RelA interaction is mediated by the first eight arm-repeats of β-Catenin: XRelA was found to bind full length β-Catenin and the fragments arm1–12 and arm1–8, but neither the N-terminal nor the C-terminal fragments (**B** and **C**: n = 2 independent experiments each). (**D**) Co-Immunoprecipitation of endogenous p65/NF-κB with β-Catenin in Wnt-stimulated REH cells. Extracts from control cells and cells stimulated with Wnt3a-containing medium were immunoprecipitated using an anti-β-Catenin antibody. Immunoprecipitates were analyzed for β-Catenin and p65/NF-κB by Western blots (n = 3 independent experiments).

To investigate the possibility of a physical interaction between Xenopus β-Catenin and Rel proteins, we first performed semi-quantitative pull-down assays with bacterially expressed, His-tagged β-Catenin and in vitro-translated, ^35^S-labelled Rel proteins (Figure 6A). With the notable exception of p50, Xenopus RelA, -2, -3, and -B proteins bound to β-Catenin. Control precipitations indicate that the Rel pull downs require β-Catenin (6His control lanes, Fig. 6A), and that β-Catenin interactions are selective under the applied conditions. While an unrelated Myc-YFP protein did not appear in the bound fraction, an Axin peptide, known to bind strongly to β-Catenin [29], was also precipitated. Comparison of the amounts of Axin and Rel proteins precipitated suggests that the various XRel proteins have a similarly strong interaction with β-Catenin.

We analyzed this interaction in more detail for RelA/p65, as this Rel family member co-operated best with β-Catenin in axis formation (see Fig. 2). In general, members of the vertebrate Rel protein family differ quite strongly in their C-terminal sequence, but share an N-terminal domain – the rel homology domain (RHD) – which is responsible for nuclear localization, DNA-binding, protein dimerization and IκB interaction [37]. We found that the RHD of RelA was sufficient for β-Catenin binding (Figure 6B), although the C-terminal portion may contribute to the interaction. The central domain of β-Catenin, containing the armadillo repeats, bound RelA preferentially, while no interaction was observed for either the N- or C-terminal peptides (Figure 6C), which harbor β-Catenin's regulatory and transactivating domains, respectively [42]. In fact, the first eight armadillo repeats were sufficient to bind RelA. The comparable efficiencies with which full-length β-Catenin, arm1–12, and arm1–8 peptides interact with RelA suggest a discrete Rel-binding interface in the anterior two-thirds of β-Catenin core domain.

To provide evidence for an in vivo interaction, we performed co-immunoprecipitation experiments with endogenous RelA/p65 and β-Catenin proteins from REH cells (Figure 6D). This is a well-characterized B-cell precursor leukemia cell line with abundant p65/RelA (lane 1), most of which is kept inactive in a complex with IκB [43]. These cells contain very low levels of β-Catenin, which are significantly increased upon stimulation of the canonical Wnt pathway by incubation with soluble WNT3a (compare lanes 1 and 2, input). β-Catenin was efficiently immunoprecipitated from the WNT3a-treated cells using the 7D11 antibody, and along with it a significant amount of p65/NF-κB protein was brought down. Unstimulated cells were used as negative controls due to their very low β-Catenin levels. The 7D11 antibody from these control cells pulled down no p65/NF-κB, demonstrating that precipitation of p65/NF-κB required activated β-Catenin. Importantly, these experiments reveal for the first time a selective physical interaction between several Rel protein family members - in particular RelA/p65 – and β-Catenin in vitro, which is observed in vivo among endogenous proteins under physiological conditions of Wnt ligand stimulation.

### β-Catenin stimulates transcription through NF-κB binding sites

**Figure 7:**
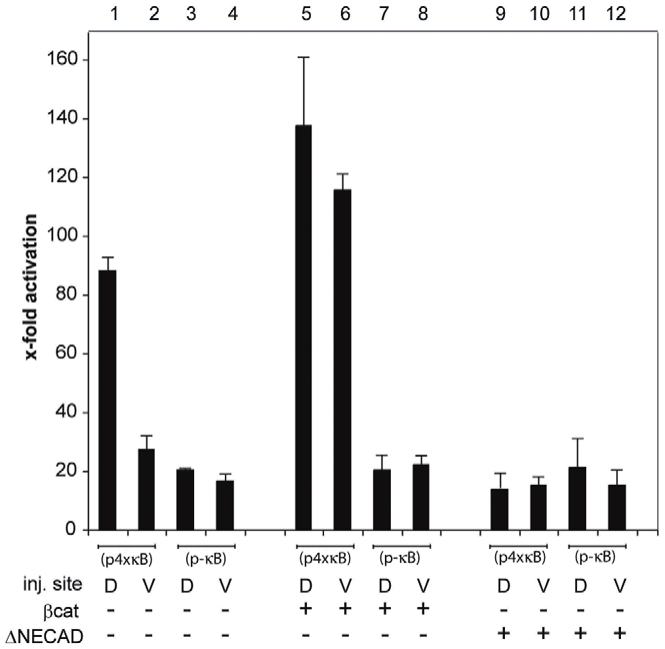
A synthetic NF-κB reporter gene is coactivated by β-Catenin. Luciferase activity at midgastrula (NF11) produced by injection of the reporter constructs pNF-κB+ (containing 4 κB binding sites) and pNF-κB− (containing none) into both blastomeres of either the dorsal or ventral side of 4-cell stage embryos. β-Catenin (25 pg; lanes 5–8) and E-cadΔE (50 pg; lanes 9–12) RNAs were co-injected with the reporter. Y-axis shows x-fold stimulation of luciferase activity over uninjected controls after normalization to the internal Renilla control.

The NFκB site in the Xnr3 promoter stimulates transcription dorsally (see Figure 5). Since the dorsal side of the embryo is defined by increased levels of soluble β-Catenin, we asked whether the transcriptional stimulation through this site is brought about by Rel/β-Catenin protein complexes. To simplify the interpretation of the results, we addressed this question with a minimal NF-κB reporter gene, consisting of four NFκB consensus sites in front of a TK promoter driving luciferase expression (p4xκB). Notably, this κB module contains no Lef/Tcf binding sites (data not shown). As a control, we used a mutated construct, in which all NF-κB sites were deleted (p-κB). When microinjected into the embryo (Figure 7), the −κB construct had comparable basal activities in dorsal and ventral sides (lanes 3 and 4). The +κB construct had about the same activity ventrally, but was about 3-fold more active dorsally (lanes 1 and 2). This bias might reflect preferential activation of NF-κB on the dorsal side, although nuclear RelA protein is reported to be distributed homogenously throughout the animal hemisphere of Xenopus blastulae [22].

In agreement with coactivation of NF-κB targets by maternal Wnt signaling, we found that overexpression of β-Catenin selectively stimulated the +κB construct, inducing it above basal level on the ventral side and hyperactivating its expression dorsally (lanes 5+6). Furthermore, coinjection of EP-cadΔE RNA together with the reporter plasmids reduced the activity of the +κB construct to basal levels (compare lanes 9+10), indicating that the dorsally enriched soluble β-Catenin levels are involved. In contrast, basal activity of the −κB construct was unaffected by either β-Catenin or EP-cadΔE RNA injections (see lanes 7, 8, 11 and 12), indicating that the observed modulation of the activity from the +κB construct depended entirely upon the κB module. Together with the Xnr3 promoter analysis, these results demonstrate that β-Catenin can function as a transcriptional coactivator of Rel/NF-κB proteins in the early frog embryo.

## Discussion

Our previous work with MyD88 has shown that signaling through a TLR/IL-1R pathway controls the expression of a specific subset of dorsally expressed genes, which are required for head formation in the frog [8]. While the identity of the extracellular ligand is currently unknown, the Xenopus genome encodes at least twenty different Toll-like receptors, which appear to be constitutively expressed in the tadpole [44]. This provides a starting point for the future identification of the TLR(s) involved in axis formation, and may help to identify also the ligand(s). In Drosophila, the Toll ligand Spätzle is activated by proteolytic cleavage through serine proteases such as Easter and Persephone [13], [45]. Our finding that enzymatically active Easter protein exerts dorsalizing activity in UV-ventralized frog embryos (Armstrong et al. 1998) is compatible with an extracellular proteolytic event to trigger nuclear translocation of Xenopus NF-κB, which has been observed in midblastula embryos [22]. In addition to potential Spaetzle homologs, which have not yet been identified in vertebrate genomes, Toll-like receptors can respond to a diverse class of endogenous ligands, including proteolytic breakdown products of extracellular matrix components [46].

The TLR/MyD88 pathway induces nuclear translocation of NF-κB through signal-dependent degradation of IκB inhibitors [47]. However, one of its components, i.e. TGF- β-activated kinase 1 (TAK1) represents a signaling nexus, which functions also downstream of MAPK, BMP and TGF-κ signaling [48]. This raises the question, which effector proteins actually mediate the TLR/MyD88 effect in Xenopus? Here, we have provided definitive proof that it is through Rel/NF-κB protein complexes. This claim rests on the following evidence. First, a transdominant IκBα variant produces the identical phenotype as inhibition of MyD88 function with regard to morphological criteria, time-dependency of interference, and transcriptional suppression of specific target genes. Secondly, we show that a NF-κB consensus motif is present in the Xnr3 promoter, one of the MyD88/IκBα-sensitive genes, and that this κB site is important for the endogenous Xnr3 expression pattern.

A corollary of the enormous diversity of biological roles assigned to the NF-κB system is that perturbation of its functions can have broad deleterious consequences. Loss of NF-κB function through targeted inactivation of critical components, such as Rel or IκB kinase (IKK-α and -β) genes, causes early embryonic lethality in the mouse, which is due to defects in neurulation, liver function and vascular epithelial cell differentiation, at least partly arising from enhanced apoptosis [49]. More informative with regard to developmental functions of the NF-κB system were studies using transdominant IκBα inhibitors, which revealed a requirement in chick limb bud morphogenesis [24], [26], differentiation of epidermal appendices [50] and Zebrafish notochord development [25].

The functions we describe here in Xenopus differ from these reports in several important aspects. We show that NF-κB activity is required for the gene expression program on the dorsal side at the late blastula stage or earlier, i.e. much earlier in development than previously anticipated. While this program has been thought to depend predominantly on maternal Wnt/β-Catenin activity [7], [51], our experiments indicate an interdependence between Wnt/β-Catenin and MyD88/IκB/Rel pathways. The observation that ΔNIκBα is active only when injected very early is strikingly consistent with the early and narrow window (16–32 cell-stage) for β-Catenin stabilization by LiCl treatment [52], and is fully in agreement with a very early interaction between these two pathways. The strongest interdependence observed for induction of secondary axis can be explained by the fact that under these conditions exogenous ΔNIκBα and β-Catenin proteins accumulate and act presumably simultaneously.

Our results suggest that β-Catenin acts on the Xnr3 promoter through two parallel mechanisms, serving as co-activator for both TFC3 and NF-κB. The synergism between animally-localized NK-κB activity and dorsal β-Catenin explains the restricted expression of Xnr3 in the BCNE [33], [53], and provides an additional example of the exquisitely fine molecular and spatial regulation of the signals controlling the patterning of the early gastrula embryo. While our interest in the xnr3 promoter arose from its NK-κB consensus binding site, it is possible that maternal Wnt/β-Catenin and MyD88/IκB/Rel pathways co-activate additional genes, potentially even before the midblastula transition [54], to unfold the dorsal gene expression program in Xenopus.

Finally, the analysis of the p4xNF-kB reporter gene demonstrates for the first time that canonical Wnt-signaling can co-activate transcription through NF-κB motifs. In principal, β-Catenin could induce other proteins, which interact with Xrel or even bind directly to the promoter of the reporter gene, to achieve this effect. While we cannot formally rule out such an indirect scenario, the simplest mechanism for our findings would involve the direct interaction between certain Rel proteins and β-Catenin, which we have demonstrated by pulldown experiments in vitro and with physiological protein concentrations upon Wnt-stimulation in vivo. The physical interaction occurs through a discrete Rel binding interface within β-Catenin's core domain and the rel homology domain of XrelA/p65.

The epistatic relationship between Wnt and TLR signaling is intrinsically complex, given that IκBα and β-Catenin are regulated by phosphorylation at similar consensus NH_2_-terminal serines and are targeted for degradation by the same E3 ubiquitin ligase complex SCF^β-TrCP^ [55], [56]. While our experiments in Xenopus consistently indicate a positive functional cooperation between NF-κB and β-Catenin, several studies with human cancer cell lines have reported that β-Catenin binds NF-κB and suppresses its DNA binding and transactivation activities. Noteworthy, β-Catenin inhibited only a subset of NF-kB target genes in some, but not all cancer cell types tested [40], [41], [57], [58]. While further experiments are needed to understand the mechanistic details, which dictate the final outcome of the interaction between canonical Wnt and TLR/IL-1R pathways, our study has provided a paradigm that it can lead to synergistic gene activation.

Cells from the animal hemisphere of the Xenopus midblastula stage represent pluripotent, uncommitted precursor cells [59]. The available data on XrelA/p65 protein distribution [22] and on NF-κB dependent dorsally expressed genes ([8]; this report) in the frog support a model, in which MyD88/IkBα-dependent activation of Rel protein complexes is involved in the earliest events of embryonic differentiation. Recent findings from mouse and human ES cells also support this notion, implicating NF-κB as either pro- or anti-differentiation factor, respectively [60], [61]. Based on our findings in Xenopus, it will be interesting to determine, whether co-regulation through Wnt/β-Catenin signaling determines NF-κB protein functions in mammalian ES cells.

## Materials and Methods

### Embryo Handling and Analysis

Xenopus embryos were generated by in vitro fertilization [14], their handling, culture, and staging followed standard procedures [62]. UV-ventralization and DAI scoring of embryos has also been described [14]. Time-dependency injections were performed subequatorially into the dorsal side of the embryo. At the 2-, 4- and 8-cell stages, two blastomeres were injected with 250 pg each, at the 16-cell stage, four blastomeres were injected with 125 pg each and at the 32-cell stage, six blastomeres were injected with 83 pg each. In some experiments, 25 pg eGFP RNA was co-injected as a lineage tracer for the injected cell progeny to confirm viability and distribution within the embryo.

### DNA Constructs and RNA Synthesis

The following cDNAs and plasmids have been described: spätzle (pCS2+spz8.19) and activated easter (pCS2+EaΔN) [14], dominant-negative MyD88 [8], XrelA [16], Xrel3 [19] and XrelB [17]. The coding regions of Xrel2 [18], human p50 [63], frog β-Catenin [64] and chick pp40 (IκBα) [18] were subcloned into pCS2^+^ by PCR. For ΔN40IκBα, amino acids 2–39 of the open reading frame were deleted by PCR. XrelAenR contains the RHD (aa 1–305) of XrelA1 [16] fused in frame to the transcriptional repressor domain of *engrailed* [65]. All new clones have been verified by sequencing. For in vitro synthesis of capped RNA transcripts by SP6 RNA polymerase, all pCS2+ derivatives were linearized with SnaB1.

### RT/PCR Analysis

Quantitative analysis of relative mRNA levels by RT-PCR was described [66]. Primers for nodal related 3, siamois [8], twin [67], histone H4 [68], snail [69], chordin, follistatin, noggin [70] have been previously described. The following primers and PCR conditions (annealing temperature/cycle numbers given in parentheses) have been used for - Xbra: forward 5′-ATGCAGTGTCCCCCCATC-3′, reverse 5′-GC AGTGCATAACTCCGAA-3′ (56°C/26); cer: forward 5′-GCTGAACTATTTGATT CCACC-3′, reverse 5′-ATGGCTTGTATTCTGTGGGGC-3′ (58°C/28); gsc: forward 5′-CTCCCTTACATGAACGTTGGC-3′, reverse 5′-TCTGAGATGAACTCTCCTTGC-3′ (55°C/30); otx2: forward 5′-CTCACATACTAACAAGCCACC-3′, reverse 5′-CCAG AAGAAGAATCTTCCAGC-3′ (58°C/28), Xnr1: forward 5′-GAGAGGCTCAGGTAT GAG-3′, reverse 5′-CTACTAGCTTTCTCTATGTC-3′ (55°C/28); Xnr2: forward 5′-GA AAAGCAGCTAAGATCC-3′, reverse 5′-CGATTGCCCACTACAACAC-3′ (55°C/27); XVent1: forward 5′-GCATCTCCTTGGCATATTTGG-3′, reverse 5′-TTCCCTTCAG CATGGTTCAAC-3′ (65°C/27); XVent2: forward 5′-TGACACTTGGGCACTGTTC TG-3′, reverse 5′-CCTCTGTTGAATGGCTTGCT-3′ (55°C/30). Triple samples, consisting of three embryo equivalents, were collected for each experimental condition (n = 3 biological repeats). PCR product amounts were highly reproducible between samples and independent experiments. To control for saturation of PCR product, each procedure was carried out simultaneously in triplicate with three sets of PCR tubes from one master sample: one set being removed 2 cycles before, one set 2 cycles after, and one set at the number of cycles which, during calibration, had been determined as optimal to measure expression levels of each gene.

### Reporter Gene Analysis

The pNF-κB-Luc vector (Cat #631904, Clontech) contains four consensus NF-κB DNA binding motifs. To generate the pMinusNF-κB-Luc vector, all κB sites were removed by BglII/Asp718 digest, blunting and religation. The Xnr3 promoter was generated by PCR from the published sequence [34] using the primers: forward 5′-AGCTCGAGCTGCAGTAGTTAAAGATACAA-3′; reverse 5′-AGAGATCTCCTC AAAAAACTGATGTACA-3′, and subcloned via XhoI/BglII into the pGL3 luciferase expression vector to produce pXnr3(wt). Site-directed mutagenesis was carried out according to the manufacturer's (Stratagene, QuikChange) instructions using the following primers: for pXnr3(−κB) 5′-CCCAGTATAAAGTCAGccggCgagaAAGACTG TACATCAG-3′, and for pXnr3(−TCF) 5′GGCTTGGATCATACAGgccAAgcgAgCcg AGGAATGAAGAATCCTG-3′. Embryos were coinjected with a TK-driven Renilla construct (pRL-TK, Promega; 25 pg/embryo) as internal control, together with the various Xnr3 luciferase constructs (50 pg/embryo). The injected embryos were cultured to stage NF 11 (midgastrula), when groups of ten embryos were collected and lysed in 200 µl 1× Lysis buffer (Dual Luciferase Kit, Promega). Luciferase activity was determined from 10 µl of the supernatant, according to the manufacturer's instructions, and quantified with a Berthold Lumat LB9501 luminometer. Relative firefly luciferase activity (RLU) was normalized with Renilla luciferase activity in cellular lysates. The presented results are calculated from triplicate samples of at least two biological repeats.

### *In vitro* pull-downs and immunoprecipitations

For in vitro pulldown assays, recombinant full length Xenopus β-Catenin and the β-Catenin fragments N-term (aa2–143), C-term (aa665–781), arm1–12 (aa144–671), arm1–8 (aa144–445), all fused N-terminally to 6xHis-Trx-S-tag (pET32b vector, Novagen), were produced in E.coli and purified according to the manufacturer's instructions. The fragment 6xHis-Trx-S-tag (110 aas) produced by the empty pET32b vector was used as negative control in all pull-down experiments. ^35^S-translated proteins were synthesized using the TNT coupled transcription/translation reticulocyte lysate system (Promega). 15 µg of recombinant proteins were mixed with 8 µl of *in vitro* translated proteins in a final volume of 50 µl Binding Buffer (20 mM Tris-HCl pH 7.5, 150 mM NaCl, 1 mM dithiothreitol) containing 0.6% Tween, and supplemented with Complete Protease Inhibitor Cocktail (Roche Molecular Biochemicals). The samples were incubated for 20 min at 37°C. 60 µl of 50% slurry S protein-agarose (Novagen) was added and the samples were incubated for 30 min at 4°C, washed 5× with 500 µl Binding Buffer (0.1% Tween). The proteins were eluted by adding 20 µl SDS-PAGE sample buffer to the beads and boiling for 5 min. Half of each sample was loaded on a 12% SDS-PAGE, and analyzed by autoradiography.

For *in vivo* coimmunoprecipitation analysis, REH cells were incubated for 4 hours at 37°C with conditioned medium of Wnt3a-secreting L-cells or with control medium of parental L-cells. Cells were lysed in IP buffer (0.5% NP40, 10% glycerol, 80 mM NaCl, 10 mM NaOH-Hepes pH7.3, 2 mM MgCl_2_, 1 mM EDTA, 50 mM NaF, 2 mM Vanadate, supplemented with Complete protease inhibitors). After a 10 min centrifugation at 13.000 g, the supernatant was incubated for 2 hrs with 4 µg 7D11 mouse monoclonal anti-β-Catenin antibody (Biomol). The samples were then cleared by centrifugation and incubated for an additional hour with 20 µl protein G beads (Sigma-Aldrich). The beads were finally washed 4 times with 500 µl IP buffer and the immunoprecipitates were eluted by boiling in SDS-PAGE sample buffer. The samples were analyzed by SDS-PAGE and Western blot using rabbit anti-β-Catenin P14L [1] and rabbit anti-p65 antibodies (sc-7151, Santa-Cruz).
